# Protein structure prediction for novel mutations in Arylsulfatase-A gene

**DOI:** 10.1186/1755-8166-7-S1-P62

**Published:** 2014-01-21

**Authors:** M Divya, S Jamal Md Nurul Jain, SR Phadke, Ratna Kishore, Mahesh Kamate, Neerja Gupta, Ashwin Dalal

**Affiliations:** 1 Diagnostics Division, Centre for DNA Fingerprinting and Diagnostics, Hyderabad, Andhra Pradesh, India

**Keywords:** Protein structure prediction, missense, frameshift, mutation

## Background

Protein structure prediction is the prediction of three-dimensional structure of a protein from its amino acid sequence. It is useful in determining the effect of a mutation on protein structure and associated function in detail. Along with the use of mutation prediction servers (Mutation taster, Polyphen etc.) protein structure prediction is an additional approach to functionally annotate genetic variants detected from different individuals.

## Methods

The X-ray structure of the P15289 protein was used for protein structure prediction of effect of mutations (PDB code 1AUK). TRANSEQ program of EMBOSS was used to obtain translated products of the deletion and insertion mutants. PyMOL and Swiss PdbViewer were used for performing structural analysis. Potential changes in overall hydrophobicity due to non-synonymous mutations were calculated using CLC workbench.

## Results

We performed protein structure prediction for six novel non-synonymous missense mutations (P180Q, Y33S, Q139K, R299P, G34E and R311P) and four frameshift mutations (c.188-189insA, c.752-753insT, c.576delC and c.445-446insT) obtained from sequence analysis of ARSA gene. The residue Q139 is in 3_10_ helix and R311 is in a β-sheet. Missense mutations at these positions affect the secondary structure of the protein. R299P mutation predicted to destabilize surrounding helical structure by hydrogen bond disruption. G34E, P180Q, R299P, and R311P showed a wide range of alterations in the overall hydrophobicity at the sites of mutation. Y33S and R311P were involved in active site modification and P180Q affecting the catalytic ability. All frameshift mutations were predicted to be leading to nonsense mediated decay.

## Conclusion

Protein structure prediction helps to provide a means of generating a plausible protein structure resulting after the mutation and understanding the effect of mutations on a protein whose effects have not been experimentally determined.

